# Habitat loss over six decades accelerates regional and local biodiversity loss via changing landscape connectance

**DOI:** 10.1111/ele.13260

**Published:** 2019-04-01

**Authors:** Zsófia Horváth, Robert Ptacnik, Csaba F. Vad, Jonathan M. Chase

**Affiliations:** ^1^ WasserCluster Lunz Lunz am See Austria; ^2^ German Centre for Integrative Biodiversity Research (iDiv) Halle‐Jena‐Leipzig Leipzig Germany; ^3^ Institute of Computer Science Martin‐Luther University Halle‐Wittenberg Halle (Saale) Germany

**Keywords:** Extinction, fragmentation, habitat connectivity, landscape connectance

## Abstract

When habitats are lost, species are lost in the region as a result of the sampling process. However, it is less clear what happens to biodiversity in the habitats that remain. Some have argued that the main influence of habitat loss on biodiversity is simply due to the total amount of habitat being reduced, while others have argued that fragmentation leads to fewer species per site because of altered spatial connectance among extant habitats. Here, we use a unique data set on invertebrate species in ponds spanning six decades of habitat loss to show that both regional and local species richness declined, indicating that species loss is compounded by habitat loss via connectivity loss, and not a result of a sampling process or changes in local environmental conditions. Overall, our work provides some of the clearest evidence to date from a longitudinal study that habitat loss translates into species loss, even within the remaining habitats.

## Introduction

Habitat loss and fragmentation are among the most important causes of decline in global biodiversity (Wilson 1988; Hanski 2005; Millennium Ecosystem Assessment 2005; Maxwell *et al*. 2016). However, there is considerable disagreement as to exactly how habitat loss and fragmentation influence species loss, and at which spatial scales species loss is observed (Fahrig 2013, 2017, 2018; Haddad *et al*. 2015; Hanski 2015; Fletcher *et al*. 2018). This disagreement dates back to questions of whether species richness is best preserved within a single large or several small habitat reserves (i.e. the SLOSS debate; Diamond 1975; Simberloff & Abele 1976) and has re‐emerged in slightly modified form in recent years. For example, there is a question as to whether habitat loss leads to species loss simply due to the process of sampling (e.g. the species–area relationship), or whether there are ecological processes occurring within the remaining habitats that lead to further biodiversity loss, for example, due to effects of habitat fragmentation and spatial connectance loss among remaining habitat patches (Fahrig 2013, 2017; Haddad *et al*. 2015; Hanski 2015). Explicit tests of these hypotheses are often confounded because the scale of sampling in which biodiversity is measured is not carefully considered, leading to a great deal of confusion as to exactly how and why biodiversity changes in the face of changing habitat amount and fragmentation (Hanski 2005; Fahrig 2017).

A clear way to test the question of whether the total amount of habitat, or the conditions within the remaining habitat, influences the patterns of biodiversity under habitat loss is to take a scale‐explicit view. It is axiomatic that habitat loss will lead to species loss at large spatial scales, simply as a result of the ubiquitous species–area relationship and its inverse, the endemics–area relationship (Kinzig & Harte 2000; Rosenzweig 2003; He & Hubbell 2011). It is less clear, however, what happens to the numbers of species in a given locality (i.e. alpha‐diversity) following the loss of surrounding habitats. The ‘habitat amount hypotheses’ and other theories predicting species losses based solely on habitat area implicitly assume that the numbers of species in a given locality of remaining habitat should be similar to the numbers in a given area of more intact habitat (e.g. Fahrig 2013, 2017). Alternatively, if habitat fragmentation (i.e. patch size and isolation) plays a strong role, the numbers of species in a given locality that is small and/or isolated will be lower than in a patch of the same size that is embedded within a more continuous network of habitats (Rybicki & Hanski 2013; Haddad *et al*. 2017).

Despite the testable predictions, evidence on the influence of habitat loss and fragmentation on local biodiversity is mixed and contentious (e.g. Haddad *et al*. 2015; Fahrig 2017, 2018; Fletcher *et al*. 2018). What is more, the vast majority of studies use spatial comparisons to infer the influence of habitat loss by comparing the numbers of species in intact habitats to those in fragmented habitats. A more direct test of the question of how habitat loss influences both local and regional species requires longitudinal data on how the number of species in a given habitat patch changes as the surrounding landscape changes, while controlling for other drivers that are simultaneously changing (e.g. habitat quality). Few such cases exist, however, and are limited to dramatic examples, such as remnants of a habitat following deforestation (Laurance *et al*. 2018) or the creation of new islands via flooding (Gibson *et al*. 2013).

**Figure 1:**
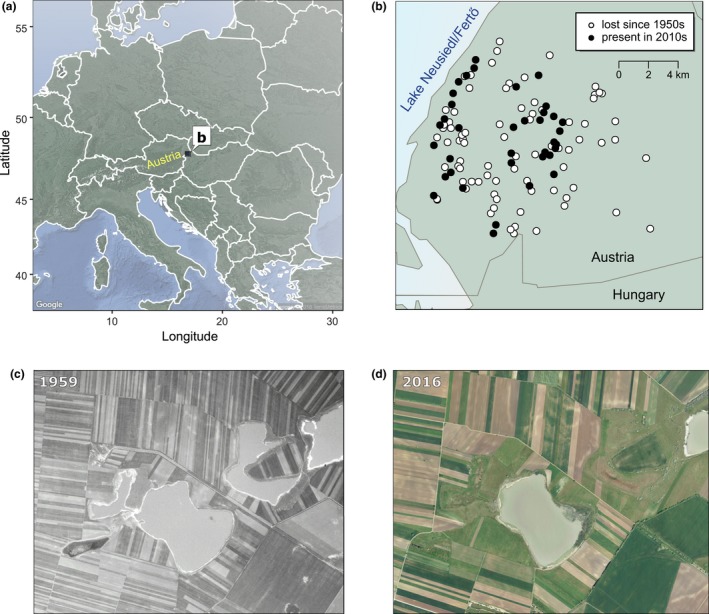
Habitat loss since the 1950s. The location of our study area in eastern Austria (a). Original habitats in 1950s (circles; b) and remaining habitats today (filled circles). Comparison of a smaller part of the region in 1950s (c; with five soda pans) and today (d; with only two remaining soda pans) on aerial photographs.

In this study, we take advantage of an exceptional data set on the local and regional diversity of invertebrate zooplankton from a natural landscape of temporary saline ponds in eastern Austria (Seewinkel region; Fig. 1) that has experienced major habitat loss over the last century. Ponds and wetlands are extremely sensitive to land use and climate change, resulting in dramatic losses in their numbers worldwide (Honegger 1981; Heath & Whitehead 1992; Wood *et al*. 2003; Curado *et al*. 2011; Davidson 2014), up to 90–100% in some regions (Honegger 1981; Hassall 2014). Likewise, the temporary saline ponds we studied in Austria, known as soda pans, have experienced a tremendous decline in total amount as a result of anthropogenic alteration in their hydroperiod, mostly due to drainage for agricultural development throughout most of the 20th century (Boros *et al*. 2013). In our study region, there were more than 110 such ponds in a 270 km^2^ region in the 1950s, a number of which were thoroughly sampled for zooplankton at that time (Löffler 1959). In 2010, at the time of our sampling, only 30 of these ponds remained (Fig. 1), indicating a c. 70% loss of habitat over six decades (corresponding to 65% loss of total surface area; Dick *et al*. 1994).

By comparing the numbers of species regionally and locally across more than half a century of habitat loss, we were able to directly evaluate how habitat loss and ensuing loss of spatial connectance, as well as change in habitat quality, influenced patterns of biodiversity in this unique ecosystem type. We explicitly evaluated whether the effect of habitat loss was due to a loss in total habitat alone, or whether there was also an influence of increasing habitat fragmentation (loss of connectance). To do so, we calculated the numbers of species that would be expected to go extinct based on the loss of the amount of habitat alone from the species–area relationship observed in 1957 and the known loss of habitat amount. We then compared this expected extinction rate to the actual observed numbers of species lost from the region more than 50 years later to determine whether species loss differed from that which would have been expected via changes in habitat amount alone. We further evaluated the relative role of multiple drivers in explaining the regional and local loss of species by comparing traits of species that went extinct from the region (habitat occupancy, habitat preference, body size), as well as changes in the characteristics of remaining habitats (change in local habitat quality, area or connectivity).

## Materials and Methods

### Zooplankton data collection and summation

In 1957, zooplankton (crustaceans and rotifers) were sampled from 55 of the 116 existing soda pans in the region at that time (Löffler 1959). By 2009, only 30 soda pans remained in the region. In 2009 and 2010, we re‐sampled all of these sites (Tóth *et al*. 2014). We randomly collected 20 litres of water from the open water of the pans, and sieved it through a plankton net with a mesh size of 30 μm. Although sample quantity or plankton mesh size was not specified in Löffler (1959), it was indicated that plankton communities were sampled similarly from the open part of the pans. As these extremely shallow habitats (average water depth is *c*. 20 cm; Boros *et al*. 2014, 2017) are well‐mixed and the open water area completely lacks macrovegetation (either emergent or submerged), samples collected from the open water should be highly comparable between the two time periods. Moreover, the study (Löffler 1959) specifically targeted both rotifers and larger crustacean zooplankton; therefore we assume that the mesh size used was appropriate for the smaller group (rotifers).

The two data sets were harmonised with regard to taxonomic changes. This included lumping species in the 2010 data set that were described between 1957 and 2010 and revising species names in the 1957 data set to match those in the 2010 data set.

**Figure 2:**
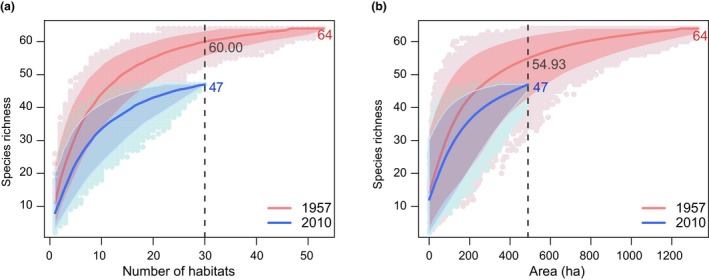
Temporal changes in regional richness based on the number (a) and area (b) of sampled habitats. Regional richness in the larger data set was predicted at a sample size of 30 ponds (number of ponds in the smaller data set from 2010; a) and at an area of 488.75 ha (size of total area in 2010; b). Confidence intervals were calculated based on quantile regression.

In 2009–2010, each soda pan was sampled during spring (2010) and summer (either 2009 or 2010), and species richness was calculated as the sum of all species observed in a given soda pan. The one exception that we did not include in our summer survey was a soda pan that has such a short hydroperiod that it does not hold water until the summer. In 1957, 39 of the 55 pans were sampled during both spring and summer (Löffler 1959), which were pooled as above, whereas the remaining 16 soda pans were only sampled in either spring or summer. Nevertheless, this limitation would have biased our results against finding a strong difference between the 1957 and 2009–2010 sampling, and thus our findings to the contrary were robust against this bias (see Figs 2 and 4). Of the 30 soda pans that remained and were sampled in 2009–2010, 24 were also sampled in 1957. We used these 24 sites for identifying potential drivers of local species loss.

### Regional egg bank analysis

In order to investigate whether the 22 species that were regionally lost between 1957 and 2010 were indeed extinct, we checked for their presence in the egg bank. For this, we collected sediment samples from 23 of the remaining 30 habitats in autumn 2013 (right after they dried). We sampled the active egg bank (the upper 3 cm of the sediment; Cáceres & Hairston 1998) along two crossed transects, thereby covering both the shorelines and the central part of each habitat, resulting in one composite sample per habitat. This dry sediment was then kept in dark at 4 °C for 4 months (for a cold trigger; Vandekerkhove *et al*. 2005). As isolation of diapausing eggs from sediment samples enhances hatching success (Vandekerkhove *et al*. 2004), we applied the sugar floating isolation protocol (Onbe 1978; Marcus 1990) with 150 g sediment. To reach the maximum number of possible hatchlings per habitat, we applied two different salinity levels for each habitat, which represented the lowest (0.5 mS/cm) and average conductivity values (3 mS/cm) of the Seewinkel pans during the 2009–2010 investigations. Samples in transparent vials were placed in an environmental chamber for constant temperature (18 °C) in a long‐day photoperiod (16 h light per day) and filled up with 150 mL medium (sodium‐carbonate dissolved in distilled water, with conductivities of 0.5 and 3 mS/cm). *Cryptomonas* was regularly added as food source to the vials, which were regularly checked for hatchlings for 30 days. Each hatchling was isolated and raised individually to a stage that allowed identification to species level.

### Habitat and landscape variables

While there is an extensive coverage of environmental conditions available for the 2010 data set, in 1957, only salinity (conductivity) was measured. We used salinity as a proxy of habitat characteristics given its close correlation with several other factors (pH, turbidity, water depth and trophic state; (Horváth *et al*. 2014, 2016) and its influence on local species richness in the studied habitats (Horváth *et al*. 2014). Moreover, salinity change is among the best indicators of degradation in these habitats (Tóth *et al*. 2014). We used the annual mean value per site for tracking habitat quality changes. To quantify the habitat preference of a given species in 1957, the annual mean value of conductivity of all occupied habitats was used.

Data on the size of individual habitats were available for 48 of the 53 soda pans sampled in 1957 (Dick *et al*. 1994). Area for the remaining five sites was calculated based on maps from Dick *et al*. (1994) and Löffler (1959), with the Google Maps Area Calculator tool (Daft Logic 2018). We used our own data for all 30 habitats in 2010 (Horváth *et al*. 2013), calculated based on georeferenced‐Google Earth satellite images with ArcGIS (ESRI 2002).

For measuring the connectivity of individual habitats, we calculated their closeness centrality index based on their position in the entire habitat network (including 30 sites in 2010 and 116 in 1957) with the R package ‘igraph’ (Csardi & Nepusz 2006). Here, a lower closeness centrality value for a given site implies high centrality (connectivity) within a network, while a temporal increase in closeness means that a site became more peripheral (more isolated) between the time periods.

### Changes in local and regional species richness

We built species accumulation relationships for 1957 and 2010 by randomly re‐sampling (*n* = 2000) both data sets (53 habitats in 1957 and 30 habitats in 2010). We fitted curves in two ways: (1) by increasing the number of habitats, and (2) by including their corresponding total area. Based on the distribution of the resampled data, we constructed confidence intervals comprising 95% of the observations by fitting the 2.5th and 97.5th percentiles for all four curves with the ‘quantreg’ package (Koenker 2018) in R.

In order to test for the relative effect of increasing area and increasing number of habitats in explaining the regional richness of species, we used GAM regression models (‘mgcv’ package; Wood 2017) adding both predictors as separate smooth terms (Appendix S1). This allowed us to assess the importance of area relative to the number of sites as predictors of species richness for every resampling step. We furthermore tested for the effect of habitat size on local species richness in both periods using linear regressions (Appendix S2).

We estimated local (within pond) species richness statistics with the ‘mobr’ package (McGlinn *et al*. 2019), as well as the variation in species composition among ponds (i.e. β‐diversity; calculated as Whittaker's β‐diversity: β = γ/α). Effect sizes ($\overset{¯}{D}$) in both indices were calculated as the average absolute difference between the two data sets (1957 and 2010), while the p‐values resulted from permutation tests with 200 permutations. We repeated the comparison of local richness for those 24 sites that were sampled at both time points (Appendix S3).

### Drivers of local and regional species loss

We tested the effect of regional rarity (ratio of occupied habitats in 1957; square root transformed) together with body size (log transformed) and habitat preference (mean conductivity value of habitats occupied in 1957) as species traits on the extinction probability of individual species over the time span of the study. For this, we used a binomial multiple regression model, with the presence or absence of a given species in 2010 as the explained variable. For calculating adjusted *R* ^2^ values for the three explanatory variables, we used the R package ‘rsq’ (Zhang 2018) on general linear models (with a binomial function) including one, two or all three predictors (to calculate all pure and shared fractions).

We examined the relative role of connectivity loss (change in closeness centrality index), change in habitat quality (change in salinity measured as conductivity) and change in local habitat size in explaining changes in local richness at 24 sites that were sampled in both time points. For this, we applied a partial linear regression using the varpart function of the R package ‘vegan’ (Oksanen *et al*. 2018). To test the significance of the three effects, we applied multiple linear regression.

## Results

When we re‐surveyed the 30 remaining ponds in 2010, we found that 22 species went extinct since the 1950s, while five species were gained, leading to a net loss of 17 species. When we sampled the egg bank of these remaining soda pans in 2013, none of those regionally extinct species were present.

By comparing species accumulation curves for both increasing numbers of habitats and increasing total area of pond surface area, we tested the hypothesis of whether the observed species losses at the regional scale resulted simply from loss of habitat, or whether there were disproportionate effects such that fewer species remained than would have been expected from habitat loss alone. For both numbers of habitats (Fig. 2a) and total pond area (Fig. 2b), we found that the accumulation curve in 2010 was below that of the curve from the 1950s, indicating an effect beyond sampling alone. Specifically, if the loss in regional diversity was just due to the loss of habitats (ponds) in the region (Fig. 2a), we would have expected a loss of four species based on rarefaction to the 30 ponds remaining in 2010 instead of the 17 observed species losses. Results were similar, but less dramatic, when we used total pond area rather than numbers of ponds (Fig. 2b). Specifically, based on total pond area, we would have expected a loss of 9 species based rarefaction to the 488.75 ha of ponds remaining in 2010 instead of the 17 observed losses. However, the confidence intervals around this latter relationship are wide, because of the inclusion of dramatically different pond sizes within the randomisation procedure.

When we directly tested the influence of total surface area of ponds relative to the number of ponds as predictors of species richness loss for each resampling step, we found a consistently stronger effect for the number of habitats than cumulative habitat area (Appendix S1). Altogether, the number of habitats outperformed total habitat area as a predictor of regional species richness in 92.2 and 97.7% of the 2000 runs for the 1950s and 2010 data respectively.

**Figure 3:**
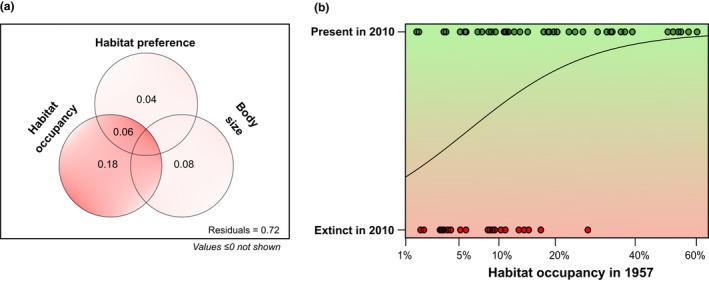
Regional extinction probability is higher for regionally rare species. In a binomial GLM model, habitat occupancy (i.e. the proportion of habitats occupied in 1957) was far more important in explaining the identity of regionally lost species than their habitat preference (salinity) or body size (a). All species that went extinct over six decades occupied less than 30% of the original habitats in 1950s (b; scale of habitat occupancy axis is square root transformed).

Species that were regionally rarer in the region during 1957 were more likely to go extinct than the species that were regionally more common (Fig. 3). In the generalised linear model (with binomial function), both regional rarity (*P* = 0.003) and body size (*P* = 0.01) had significant effects on extinction probability (p‐value for habitat preference: 0.09). However, regional rarity had an overall much higher pure effect (*R* ^2^_adj_ = 0.18) than the other predictors (*R* ^2^_adj_ = 0.04 for habitat preference and *R* ^2^_adj_ = 0.08 for body size).

**Figure 4:**
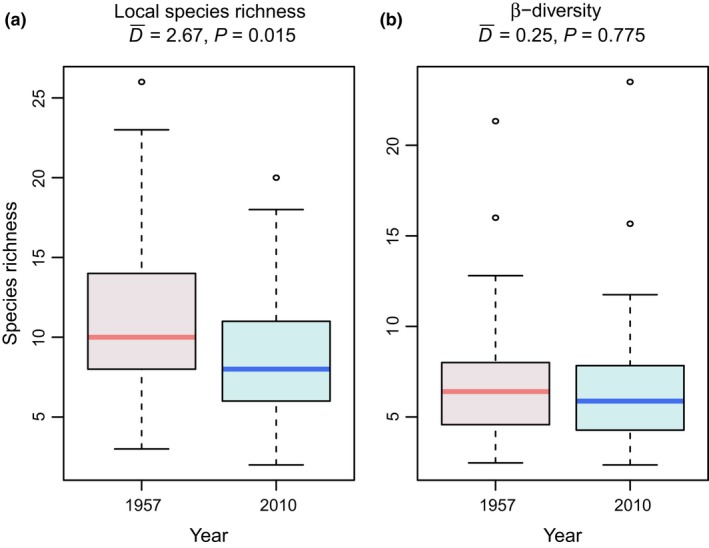
Temporal changes in local species richness (a) and β‐diversity (b). Effect size ($\overset{¯}{D}$) gives the average absolute difference between the two time periods.

Mirroring the result that there were fewer species regionally in 2010 than would have been expected simply as a result of habitat loss, we also found significantly fewer species locally (in each pond) in 2010 when compared to the 1950s. Specifically, there was an average loss of *approx*. three species per locality over the time period (Fig. 4a). When repeating the comparison of local richness for only those 24 sites that were sampled at both time points, we found the same pattern as in the whole data set (with significantly fewer species in 2010 than in 1957; Appendix S3). We found no change in β‐diversity between the two time points (Fig. 4b).

**Figure 5:**
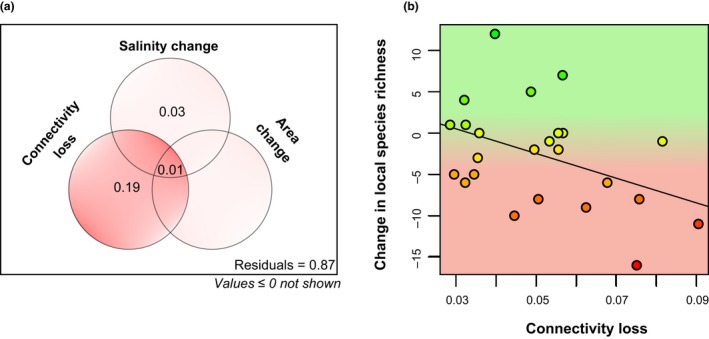
Drivers of changes in local species richness. Habitat loss via connectivity loss had a stronger effect on changes in local species richness than salinity or area change (a). Within ponds, the change in local species richness over the time period was negatively related to the amount of connectivity a given pond lost (b).

Although ponds did shrink over the time period (see Fig. 1 for an example), we found no relationship between pond area and local species richness in either period (Appendix S2). Ponds on average also became more saline (measured via conductivity) from the 1950s to 2010 (with a mean increase of 0.77 mS/cm in the conductivity of the 24 extant ponds, but with local changes ranging from a decrease of 5.02 mS/cm to an increase of 8.50 mS/cm). At the local scale, connectivity loss of a given habitat had a strong role in explaining changes in the number of species per pond (*P* = 0.028), while the effects of salinity change (*P* = 0.21) and local area change were not significant (*P* = 0.62; Fig. 5a). Figure 5b illustrates the strong negative effect of the reduction in the total number of soda pans in the metacommunity, and resultant loss of habitat connectivity on local richness (pure effect: *R* ^2^_adj_=0.19).

## Discussion

After decades of intense interest, it may seem as though we should have answered the question of how habitat loss and fragmentation influences biodiversity across scales. But recent dialog suggests the question is far from resolved (Fahrig 2015, 2017; Haddad *et al*. 2015, 2017; Hanski 2015). Indeed, ours is one of the few studies that is able to explicitly capture the influence of habitat loss and connectivity on both regional diversity (due to sampling and fragmentation effects) and local diversity (due solely to fragmentation effects) in a longitudinal study (see also Gibson *et al*. 2013; Laurance *et al*. 2018). Overall, our results support the view that the number of species that can co‐occur in the region and in a given locality is strongly influenced by the landscape in which a local patch is embedded.

The null expectation is that species would go extinct from the region when habitats are lost simply because of the geometric arrangement of habitat loss and consequent loss of species that were locally endemic to those habitats (e.g. Kinzig & Harte 2000; He & Hubbell 2011; May *et al*. 2019). This was clearly not the case in our study, as we found that most (18 of the 22) of the species we determined to be regionally extinct were present in ponds in the 1950s that still existed in 2010. As a consequence, the reductions in regional and local species richness are more likely a result of changes either to the local environment or to the regional landscape in which individual ponds are embedded.

Among the potential regional effects of habitat loss on diversity loss beyond simply losing the habitats of endemic species include: (1) reductions in the total numbers of ponds left in the landscape, (2) reductions in the total surface area of ponds left in the landscape or (3) reductions in the habitat connectivity (and likely dispersal) among the ponds remaining in the landscape. If species extinctions were due simply to loss of total numbers of habitats, or total surface area of habitats, we would have expected the species accumulation curves from the historical and recent data sets to largely overlap. Instead, we found that when calculated with both numbers of ponds and total surface area, more species went extinct from the habitats than would have been expected from habitat loss alone. The results for total surface area, however, are less dramatic, and somewhat more equivocal because of the large errors associated with randomisations when combining ponds with dramatic differences in size. Nevertheless, our GAM analysis supports the notion that species loss in these ponds was more strongly influenced by the number of ponds, rather than their total surface area. This is also supported by the fact that we observed a significant loss of species at the within‐pond scale, but that this effects also seemed unrelated to changes in pond area over time.

Our observed results of reductions in regional and local richness could have also emerged if local conditions changed such that certain species were disfavoured and driven locally and regionally extinct. While we do not have a full comparison of changes in local conditions from these ponds between the 1950s and 2010 sampling periods, we were able to compare salinity which also serves as an important proxy for other environmental factors (Horváth *et al*. 2014, 2016) and an indicator of habitat degradation in these ponds (Tóth *et al*. 2014). Like changes in habitat area, however, we found no influence of changes in salinity on local species richness.

Given the fact that our observed regional and local reductions in species richness appear to be unrelated to sampling effects due to lost habitat, or to changes in local environmental conditions, we argue that the remaining hypothesis, altered spatial processes via changed habitat connectivity, is the most likely. This implies that most species went extinct from local habitats not as an immediate sampling effect with habitat loss, but rather as a delayed effect. The loss of a large proportion of ponds in our study area over six decades resulted in reduced habitat connectivity, which could have shifted the colonisation‐extinction dynamics, resulting in a lower diversity. Our results comply with this metacommunity perspective, because regionally rare taxa were especially likely to be lost, which are typically more sensitive to connectivity loss (Hanski 1982; Angermeier 1995; Cadotte & Lovett‐Doust 2007). Indeed, at local sites, we found a strong relationship between the change in connectivity of a given pond in the landscape and its loss of local diversity, whereas changes in habitat quality (salinity) and local habitat size had negligible influence.

Our results emphasise that spatial processes, rather than simply sampling effects and changes in local habitat characteristics, can play a critical role in the conservation of biodiversity in landscapes. While the importance of spatial processes in maintaining local and regional diversity has been suggested for decades in both basic (Ricklefs 1987; Leibold & Chase 2017) and applied studies (Hanski 2005), definitive evidence has been elusive. In the context of habitat loss, this has led to considerable disagreement as to how important these spatial processes are for understanding and forecasting species diversity loss as habitats are lost (Haddad *et al*. 2015; Fahrig 2017). For example, scenarios of biodiversity loss with habitat loss that ignore spatial processes such as the disruption of metacommunity colonisation‐extinction dynamics (e.g. He & Hubbell 2011; Fahrig 2015) can strongly underestimate losses that actually occur when spatial processes are altered (e.g. Rybicki & Hanski 2013). In our case, the increase in extinction rate relative to that which would have been expected from habitat loss alone was a strong indication of fragmentation effects (Fahrig 2013; Haddad *et al*. 2017) via the disruption of spatial processes that combine with local factors to maintain diversity in this metacommunity.

Habitat loss and fragmentation due to human activities continue to accelerate (Turner *et al*. 1990; Tilman *et al*. 2001; Pullin 2002; Davidson 2014; Taubert *et al*. 2018), and there is no doubt that biodiversity is changing as a result (Wilson 1989; Brooks *et al*. 2002; Hanski 2005; Barnosky *et al*. 2011; Rybicki & Hanski 2013). Exactly how this biodiversity is changing, and at which scales, however, have been the focus of an ongoing unresolved debate (Fahrig 2015, 2017, 2018; Haddad *et al*. 2015, 2017; Hanski 2015; Fletcher *et al*. 2018). Our study provides some of the clearest evidence from a longitudinal study (over more than 50 years) that habitat loss leads to both the loss of species at the regional scale, but also at the local scale, indicating a clear disruption of the spatial processes (i.e. connectance) that maintain local diversity. Especially in the context of aquatic habitats, which continue to be eliminated and spatially fragmented via a number of land use changes (Davis & Froend 1999; Johnston 2013; Davidson 2014), this result is critical for predicting how and why biodiversity has changed, and will continue to change into the future.

## Authorship

ZH, JMC and RP designed the study. ZH performed the analyses, with suggestions from JMC, RP and CFV. ZH and CFV harmonised the data sets and conducted the regional egg bank analysis. ZH and JMC wrote the first draft, after which all the authors contributed to improving the manuscript.

## Data accessibility statement

Data supporting the results are publicly available on Figshare Repository: https://doi.org/10.6084/m9.figshare.7823726.
