# Supplementary material for: Habitat loss over six decades accelerates regional and local biodiversity loss via changing landscape connectance
Source: Ecol Lett. 2019 Apr 1;22(6):1019–27. doi: 10.1111/ele.13260 (PMC6518933; doi:10.1111/ele.13260)

Appendix S1. Relative effect of increasing area and increasing number of habitats in explaining regional species richness based on a GAM model (with both predictors as separate smooth terms). Coloured points indicate the observed species richness, while contours show the predicted species richness based on the respective GAM model for the 1957 (a) and 2010 datasets (b). Distribution of p values for both predictors in the GAM models are summarized on boxplots for each of the 2000 permutations. The red line (c and d) indicates p=0.05.


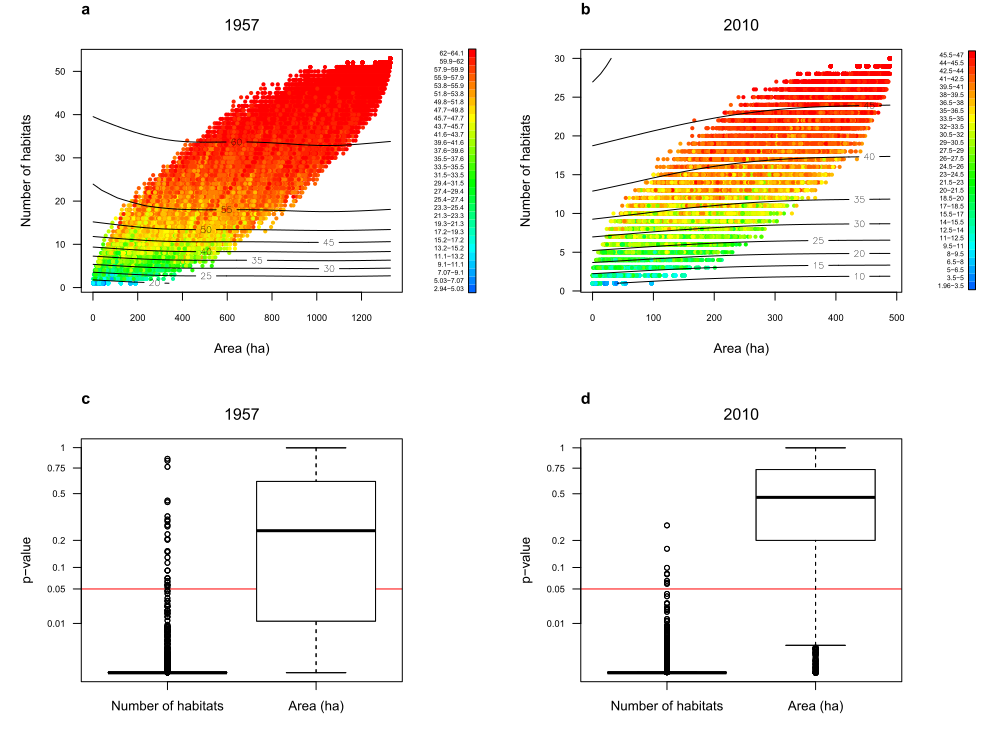


Appendix S2. Local species richness was not related to habitat area in either time point (linear model with log-transformed area; R^2^=0.002 and p=0.77 in 1957; R^2^=0.05 and p=0.24 in 2010).


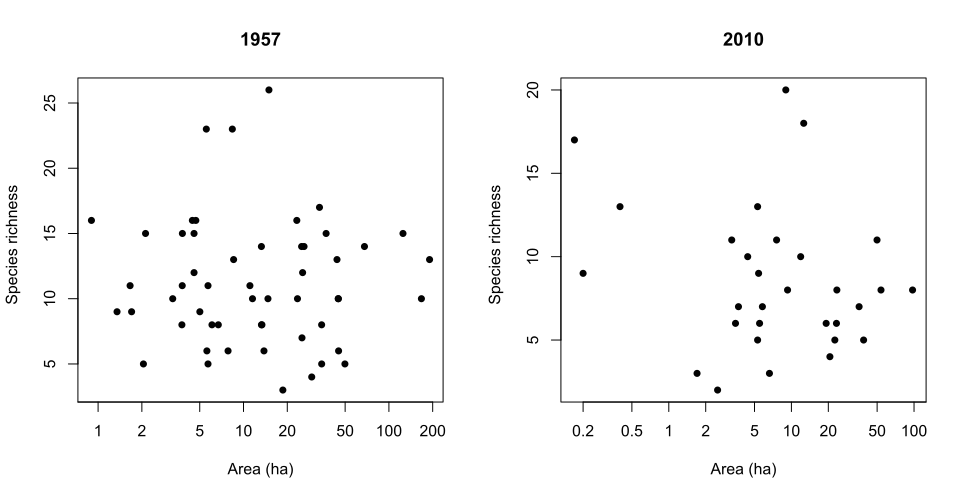


Appendix S3. Temporal changes in local species richness at the 24 sites where observations are available from both 1957 and 2010. Effect size ($\bar{\boldsymbol{D}}$) gives the average absolute difference between local diversity in the two time periods.


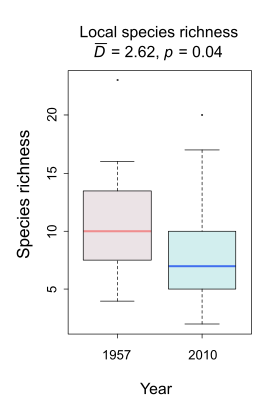

Supplement: Supplementary file 1 [file ELE-22-1019-s001.docx]
